# Mimitin – a novel cytokine-regulated mitochondrial protein

**DOI:** 10.1186/1471-2121-10-23

**Published:** 2009-03-31

**Authors:** Paulina Wegrzyn, Stephen J Yarwood, Nathalie Fiegler, Monika Bzowska, Aleksander Koj, Danuta Mizgalska, Stanisław Malicki, Magdalena Pajak, Aneta Kasza, Neli Kachamakova-Trojanowska, Joanna Bereta, Jacek Jura, Jolanta Jura

**Affiliations:** 1 Department of Cell Biochemistry, Faculty of Biochemistry, Biophysics and Biotechnology, Jagiellonian University, Krakow, Poland; 2 Faculty of Biomedical and Life Sciences, University of Glasgow, UK; 3 National Research Institute of Animal Production, Department of Biotechnology of Animal Reproduction, Balice, Poland

## Abstract

**Background:**

The product of a novel cytokine-responsive gene discovered by differential display analysis in our earlier studies on HepG2 cells was identified as mimitin – a small mitochondrial protein. Since proinflammatory cytokines are known to affect components of the respiratory chain in mitochondria, and mimitin was reported as a possible chaperone for assembly of mitochondrial complex I, we looked for the effects of modulation of mimitin expression and for mimitin-binding partners.

**Results:**

By blocking mimitin expression in HepG2 cells by siRNA we found that mimitin has no direct influence on caspase 3/7 activities implicated in apoptosis. However, when apoptosis was induced by TNF and cycloheximide, and mimitin expression blocked, the activities of these caspases were significantly increased. This was accompanied by a slight decrease in proliferation of HepG2 cells. Our observations suggest that mimitin may be involved in the control of apoptosis indirectly, through another protein, or proteins. Using the yeast two-hybrid system and coimmunoprecipitation we found MAP1S among proteins interacting with mimitin. MAP1S is a recently identified member of the microtubule-associated protein family and has been shown to interact with NADH dehydrogenase I and cytochrome oxidase I. Moreover, it was implicated in the process of mitochondrial aggregation and nuclear genome destruction. The expression of mimitin is stimulated more than 1.6-fold by IL-1 and by IL-6, with the maximum level of mimitin observed after 18–24 h exposure to these cytokines. We also found that the cytokine-induced signal leading to stimulation of mimitin synthesis utilizes the MAP kinase pathway.

**Conclusion:**

Mimitin is a mitochondrial protein upregulated by proinflammatory cytokines at the transcriptional and protein levels, with MAP kinases involved in IL-1-dependent induction. Mimitin interacts with a microtubular protein (MAP1S), and some changes of mimitin gene expression modulate activity of apoptotic caspases 3/7, suggesting that this protein may indirectly participate in apoptosis.

## Background

Tissue injury initiates complex inflammatory reactions known as the acute phase response in which the principal role is played by cytokines such as IL-1, TNF and IL-6. In the liver these cytokines, particularly IL-6, drastically alter the pattern of synthesized cellular and secreted proteins [1,2]. Established cell lines of liver origin (e.g. HepG2 cells) represent a useful model for studying the regulation of liver-specific gene expression. In a recently published report [3] we employed differential display analysis to monitor changes in the transcript profile of HepG2 cells stimulated with IL-1, IL-6 and a mixture of both cytokines. We identified over 80 genes responding to these cytokines and encoding several proteins of known structure and function. Additionally, we found some 40 cytokine-sensitive transcripts coding for unknown or poorly characterized proteins. One of those genes coding for a 20 kDa polypeptide was selected for further detailed characterization.

During those studies we concluded that the analyzed sequence corresponds to a gene described recently by Tsuneoka and co-workers [4] and induced in various human tumor cells overexpressing c-Myc. The protein product of this gene was named mimitin (*Myc-induced mitochondrial protein*) since it was localized in mitochondria and contained an ATP/GTP binding motif as well as a domain called Complex1_17_2 kDa. These data strongly suggested that mimitin may be involved in ATP metabolism in mitochondria. In agreement with the postulated regulatory role of c-Myc in the expression of mimitin, a specific c-Myc binding site was identified in the promoter region of mimitin gene. Further analyses carried out by independent research groups [5,6] suggest that mimitin plays the role of a molecular chaperone for the assembly of mitochondrial complex I.

In the present paper, we report that the mimitin gene is activated by the proinflammatory cytokines IL-1 and IL-6, and we describe the temporal pattern of cytokine response as well as identification of the signalling pathways involved. We also compare the abundance of mimitin transcript in different human tissues and analyze the significance of mimitin for cell proliferation and cell response to apoptotic signals.

## Results

### Cytokine-induced changes in the expression of mimitin gene

**Figure 1:**
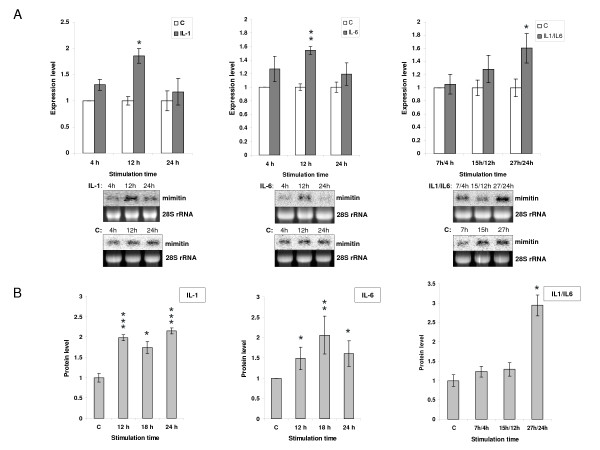
**Mimitin gene expression in HepG2 cells stimulated with IL-1, IL-6 or with both cytokines**. Cells were stimulated with the cytokines and the mimitin transcript level was evaluated by Northern blotting (A). The data are presented relatively to the mimitin transcript level in unstimulated control cells harvested after 4, 12, and 24 h. A lower panel shows representative results from Northern blot hybridization and ethidium bromide-stained 28S rRNA. Changes in the mimitin protein level estimated by ELISA were calculated relatively to unstimulated control cells (B). Vertical bars indicate the values of standard error of three independent experiments (*p < 0.05, **p < 0.02, ***p < 0.01).

The mimitin transcript was initially detected by us [3] by differential display analysis in HepG2 cells stimulated with IL-1. To study the importance of proinflammatory cytokines in mimitin gene expression HepG2 cells were stimulated with IL-1, IL-6 or a mixture of both cytokines. Changes in gene expression were evaluated at the transcript and protein levels (Fig. 1A and 1B). In Northern blot analysis the densitometric values of bands corresponding to mimitin transcript were measured for control (unstimulated) cells and cells stimulated with IL-1 or IL-6. In case of cytokine mixture, cells were prestimulated with IL-1 and then stimulated with IL-6 to simulate the physiological cascade of events. In all cases exposure to cytokines led to an increase in mimitin mRNA abundance. The highest transcript level observed after 12 h of stimulation with IL-1 or IL-6 exceeded control levels by 1.9 and 1.5 times, respectively (Fig. 1A). In the case of the two cytokines combined we observed 1.6-fold up-regulation of mimitin mRNA after 27/24 h of cytokine exposure (Fig. 1A).

To evaluate changes in protein level polyclonal antisera of high titre were raised in rabbits against recombinant mimitin. The antibodies generated specifically recognized recombinant as well as endogenous mimitin in Western blot analysis. For accurate serial measurements ELISA tests were carried out and the obtained results compared with the Northern blotting data. As shown in Fig. 1B, a 2-fold increase of mimitin level occurs after 12 h of stimulation with IL-1, or after 18 h of stimulation with IL-6. A sustained increased level of mimitin is still seen after 24 h stimulation with IL-1 alone or with the IL-1/IL-6 mixture.

The obtained results indicate that stimulation of HepG2 cells with the mixture of cytokines leads to a more prolonged expression of the mimitin gene than stimulation with a single cytokine, the effect being manifested at the levels of both mRNA and protein.

### Induction of mimitin by IL-1 involves MAP kinases but not the NFκB pathway

**Figure 2:**
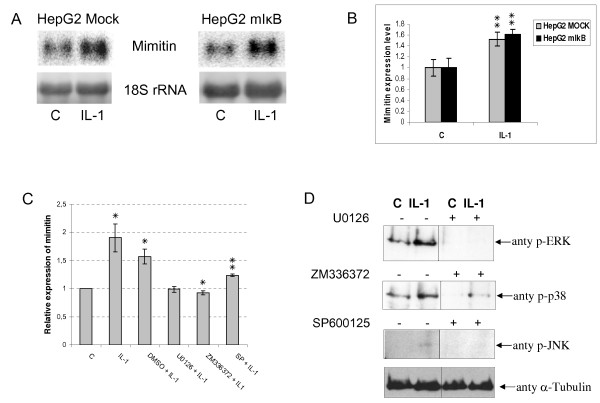
**Signalling pathway engaged in IL-1-dependent activation of mimitin gene**. A. Representative Northern blots: HepG2 cells stably transfected with retroviral vector pCFG5-IEG2 encoding nondegradable mutant of IκBα, and cells with an empty vector (mock-control) were stimulated with IL-1 (12 h) and the level of mimitin transcript was measured. Results obtained for both types of cells were normalized to 18S rRNA level. B. The graph represents data calculated from three independent Northern blot experiments. Error bars indicate the values of standard deviation (**p < 0.02). C. HepG2 cells were pretreated with: 10 μM U0126, 10 μM SP600125, or 10 μM ZM336372 for 30 min and then stimulated with 15 ng/ml IL-1 for 12 h. The level of mRNA coding for mimitin was estimated in comparison to control (unstimulated cells) by real-time PCR. Error bars indicate standard deviation from three independent experiments (*p < 0.05, **p < 0.02). D. Western blot analysis confirming activity of inhibitors of MAPK kinases. HepG2 cells were pretreated for 30 min with inhibitors (as in Fig. 2C), stimulated with 15 ng/ml IL-1 for 10 min and then cell lysates were analyzed by Western blotting with anti-P-Erk, anti-P-JNK and anti-P-p38 antibodies. The blot was later stripped and reprobed with an α-tubulin antibody (*bottom panel*) to ensure equal loading.

To investigate the role of NFκB signalling pathway in activation of mimitin gene by IL-1 we used a cell line stably transfected with retroviral vector pCFG5-IEG2 encoding a non-degradable mutant of the inhibitor of NFκB (mIκBα). Cells stably transfected with the empty vector served as a control. The control and mIκBα-expressing cells were stimulated with IL-1 for 12 h. To monitor the level of mimitin transcript Northern blot analysis was carried out. As shown in Fig. 2A and 2B, the level of mimitin transcript in HepG2 cells expressing mIκBα and stimulated with IL-1 is the same as it is in case of IL-1 stimulated control. Thus, the NFκB signalling pathway appears not to be involved in the expression of IL-1-dependent activation of mimitin gene. It is known, however, that another signalling pathway important in the expression of IL-1-regulated genes involves MAP kinases. To check if this pathway is engaged in mimitin regulation three potent, selective, pharmacological inhibitors of MAPKs cascade were used: U0126 for ERK, SP600125 for JNK, ZM336372 for p38 The specificity of each inhibitor was verified by Western blotting with antisera against phosphorylated forms of individual kinases (Fig. 2D), and a lack of cytotoxic effects was confirmed by the MTT assay (data not shown). As shown in Fig. 2C all these inhibitors abolished the IL-1-induced elevation of mimitin mRNA level, confirming the involvement of MAP kinases in the stimulation of mimitin gene expression by IL-1.

### Expression of mimitin in human tissues

**Figure 3:**
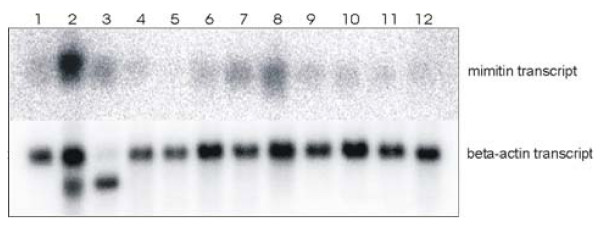
**Abundance of mimitin transcripts in human tissues**. ^32^P-labelled molecular probe for mimitin or beta actin transcript (control) was used for hyridization with commercial membrane loaded with 2 μg of mRNA isolated from 12 different human tissues: 1. brain, 2. heart, 3. skeletal muscles, 4. colon, 5. thymus, 6. spleen, 7. kidney, 8. liver, 9. small intestine, 10. placenta, 11. lung, 12. peripheral blood leukocytes. For mimitin gene, a single trasncript of 640 nt is detected; for beta actin a single 2.0 kb band is present in all lanes. A 1.8 kb actin isoform is visible in the heart and skeletal muscle lanes.

A commercial blot with mRNA isolated from a number of normal human cells, organs and tissues was used to estimate the pattern of mimitin expression at the mRNA level. The probe corresponding to the whole coding sequence of mimitin gene recognized a single transcript of approximately 640 nt. Among the tissues examined, the mimitin mRNA level was the highest in the heart, with considerably lesser amounts found in the liver, skeletal muscles and kidney. Only traces were detected in the other tissues investigated (Fig. 3).

### Subcellular localization of mimitin

**Figure 4:**
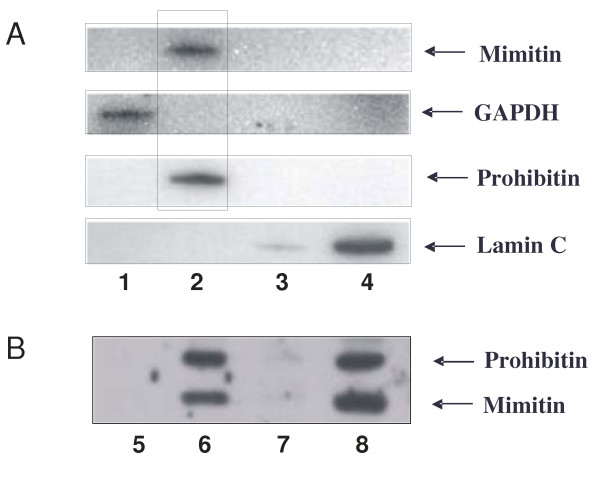
**Subcellular localization of mimitin**. Subcellular fractionation of HepG2 cells was done with Qproteome Kit (A) and the obtained cytosolic (lane 1), membrane (lane 2), nuclear (lane 3) and cytoskeletal proteins (lane 4) were checked for the presence of appropriate markers with monospecific antibodies. Western blotting showed the presence of mimitin exclusively in the membrane fraction. In additional experiments cytosolic (lane 5), mitochondrial (lane 6), microsomal (lane 7) and crude nuclear fraction containing unlysed cells (lane 8), were isolated (see Materials and Methods). This result confirmed the presence of mimitin solely in mitochondria.

To determine the subcellular localization of mimitin the Qproteome Kit for cell compartment fractionation was used. Protein extracts were isolated from HepG2 cells according to manufacturer's procedure, separated on polyacrylamide gel and analyzed by Western blotting with monoclonal or polyclonal antibodies specific for mimitin. Commercial anti-laminin, anti-GAPDH and anti-prohibitin polyclonal antibodies were used to detect markers of typical subcellular fractions. The analysis revealed that mimitin is co-localized with prohibitin in the membrane fraction (Fig. 4A). This fraction consists of plasma and organelle membranes (except for the nuclear envelope). In order to determine the localization of mimitin in more detail the procedure of Ndubuisi et al. was used to isolate the mitochondrial fraction [7]. Analysis of this fraction confirmed mitochondrial association of mimitin (Fig. 4B), in full agreement with the data of Tsuneoka et al. [4].

### Identification of mimitin-binding proteins

The results of experiments described below suggested that mimitin, being a mitochondrial protein, might participate in various pathways of cellular metabolism and initiation of apoptosis. This participation could be indirect, via a partner protein. As the first step in the identification of mimitim partners we used yeast two-hybrid screening and the Matchmaker human foetal brain cDNA library. From 31 selected clones seven positive clones were isolated of which four encoded microtubule associated protein 1S (MAP1S), one encoded stathmin-like 2 protein and the remaining two had no similarity to any cDNA in the NCBI database. MAP1S protein was chosen for further analysis because Liu and co-workers [8] showed that when MAP1S protein accumulates in cells, it appears to be localized on mitochondria and induces distinct perinuclear aggregates of mitochondria. Moreover, it was shown that MAP1S interacts with mitochondrial NADH dehydrogenase I and cytochrome c oxidase I [9].

**Figure 5:**
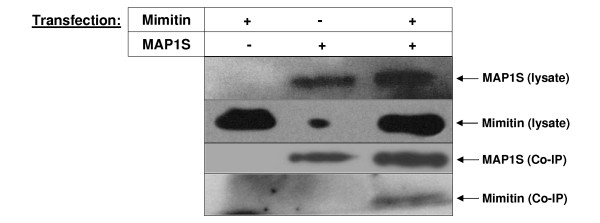
**Coimmunoprecipitation of mimitin with MAP1S**. In HepG2 cells MAP1S with c-Myc tag and mimitin genes were overexpressed. The coimmunoprecipitation analysis was performed with ProFoundTM c-Myc Tag IP/Co-IP Kit. Proteins present in cells extracts and eluates were detected in Western blot with antibodies specific for Mimitin and specific for Myc tag in case of MAP1S.

We carried out a coimmunoprecipitation assay to investigate whether mimitin associates with MAP1S *in vivo*. Vectors encoding Myc-tagged MAP1S with its transactivator and mimitin were transfected separately or together into HepG2 cells. Protein lysates were loaded on a column with antibodies for c-Myc and specific eluates were then subjected to Western blotting with either anti-mimitin or anti-Myc antibodies. As shown in Fig. 5, signals for MAP1S are visible with c-Myc antibodies in eluates prepared from HepG2 cells overexpressing only MAP1S-c-Myc or MAP1S-c-Myc with mimitin. A signal for mimitin is seen only for the eluate from HepG2 cells overexpressing both proteins (Fig. 5). This indicates that mimitin does in fact form a complex with MAP1S *in vivo *which confirms the yeast two-hybrid data.

### Effects of mimitin level on cell viability

To investigate the role of mimitin on cell viability we analysed the influence of changes of mimitin gene expression on HepG2 cell proliferation and susceptibility to apoptosis. For effective knockdown of mimitin gene, HepG2 cells were transfected with two different siRNAs (A and B) in two concentrations (50 and 100 nM). As a positive control siRNA specific for GAPDH was used, whereas a siRNA with a scrambled sequence served as a negative control. Additional control (blank) was obtained by treatment of cells with the transfection reagent siPORT™ *NeoFX*™ alone. The efficiency of knockdown was evaluated 72 h post transfection by measuring mimitin level by Western blotting. We found that cell treatment with mimitin-A-specific siRNA led to an almost complete disappearance of this protein from the cells, while siRNA-mimitin-B only slightly reduced mimitin level (data not shown). The silencing effect was specific, since there was no reduction of mimitin level in the cells treated with GAPDH siRNA, negative control siRNA or transfection reagent alone. Based on preliminary results, mimitin-A siRNA at 50 nM was selected for further knockdown experiments.

**Figure 6:**
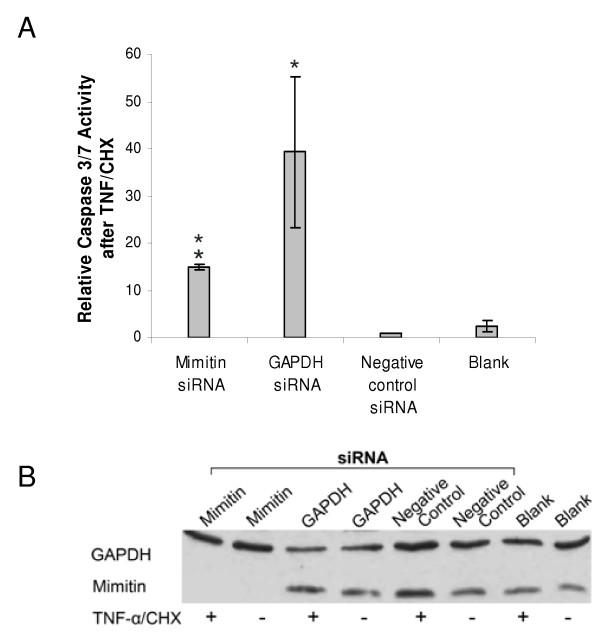
**Effect of mimitin gene silencing on caspase 3/7 activities**. HepG2 cells were transfected with mimitin-siRNA, GAPDH-siRNA, negative control siRNA, or transfection reagent (siPORT NeoFX) (blank). Apoptosis was induced 72 h post transfection by treatment of cells with TNF/CHX for 5 h. Caspase 3/7 activity was measured using Caspase-GloTM3/7 assay kit. A. Changes in caspase activity in cells with TNF/CHX-induced apoptosis were compared to cells treated with TNF/CHX and transfected with a negative control siRNA (assumed as equal 1). In each case the value of caspase 3/7 activity after TNF/CHX treatment was divided by the value of basal activity of non-stimulated cells. Values represent the mean of three independent experiments with error bars showing standard error (*p < 0.05). B. Representative Western blot analysis with 20 μg protein from caspase activity assay using anti-GAPDH and anti-Mimitin antibodies. Samples marked (+) were stimulated with TNF/CHX while samples marked (-) were not stimulated.

The mimitin gene was silenced in HepG2 cells using the established parameters and then caspase 3 and 7 activities were assayed, since they are known to be involved in programmed cell death [10-12]. The knockdown of mimitin gene had no effect on caspase 3 and 7 activities. This indicated that the level of mimitin has no direct influence on the basic activities of these caspases; however, it could still modulate them once they had been induced by a pro-apoptotic signal. Indeed, when we first induced apoptosis by treating the cells with TNF/CHX and then determined these caspases we found a striking effect of mimitin. HepG2 cells treated with siRNA specific for mimitin (mimitin-A) showed approximately 15 fold increase in the activity of caspases in comparison to negative control (Fig. 6A). Mimitin silencing was confirmed by Western blot analysis (Fig. 6B). However, treatment of cells with a GAPDH-specific siRNA had an even stronger effect on caspase activities (38-fold increase), although the protein level of GAPDH was only slightly reduced (Fig. 6B). The effects of both transfections show statistically significant differences in comparison to the negative control, with *p *< 0.02 for the mimitin-specific siRNA and *p *< 0.05 for GAPDH specific siRNA. The result obtained in HepG2 cells with the GAPDH-specific siRNA is not surprising because some reports indicate that silencing of the gene coding for GAPDH in tumour cells decreases the rate of glycolysis, inhibits cell proliferation and eventually leads to apoptosis [13,14].

We conclude that although mimitin has no direct influence on the basal activities of caspases 3/7, changes of its expression may profoundly affect these enzymes in the course of TNF/CHX-induced apoptosis.

**Figure 7:**
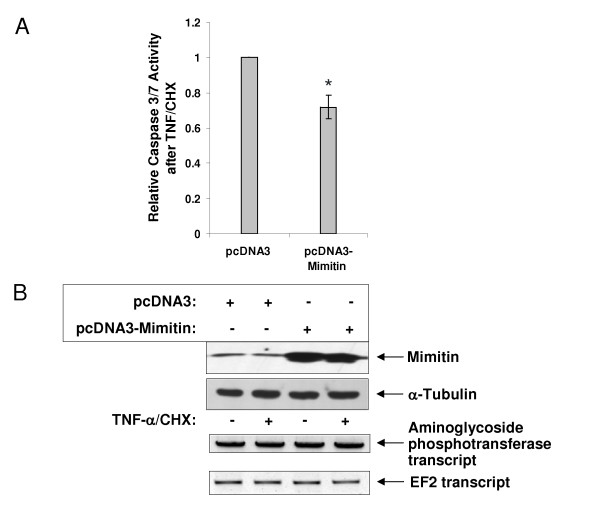
**Overexpression of mimitin decreases caspase 3/7 activities**. HepG2 cells were transfected either with pcDNA3 empty vector, or pcDNA3mim and then not-treated (control) or treated with TNF/CHX. Caspase activity was determined using Caspase-GloTM3/7 reagent. A. Graph presents changes in the relative changes in caspase 3/7 activity. In each case the value of caspase 3/7 activity after TNF/CHX treatment was divided by the value of basal activity of nonstimulated cells. Caspase activity in cells overexpressing mimitin was compared to cells transfected with an empty vector pcDNA3 (assumed as equal 1). The values represent the mean of 3 independent experiments ± SD with *p < 0.05. B. Western blot confirming overexpression of mimitin in cells transfected with pcDNA3mim. The blot was later stripped and reprobed with an α-tubulin antibody (*lower panel*) to ensure equal loading. RT-PCR results show transcript level for neomycin resistance gene (exogenous gene in pcDNA3) as a control of transfection efficiency and transcript level for elongation factor 2 (endogenous gene) as a control of RNA amount in tested samples.

The importance of mimitin in regulation of caspase 3/7 activities during apoptosis was confirmed by overexpression of mimitin in HepG2 cells treated with a mixture of TNF and CHX. HepG2 cells transfected with the pcDNA-mimitin vector and treated with TNF/CHX showed a 2-fold decrease of caspase 3/7 activity compared to control (Fig. 7). This result is in a full agreement with the data obtained after knockdown of mimitin gene expression. Silencing of mimitin gene in cells with induced apoptosis increases caspase 3/7 activities, whereas its overexpression gives an opposite effect.

**Figure 8:**
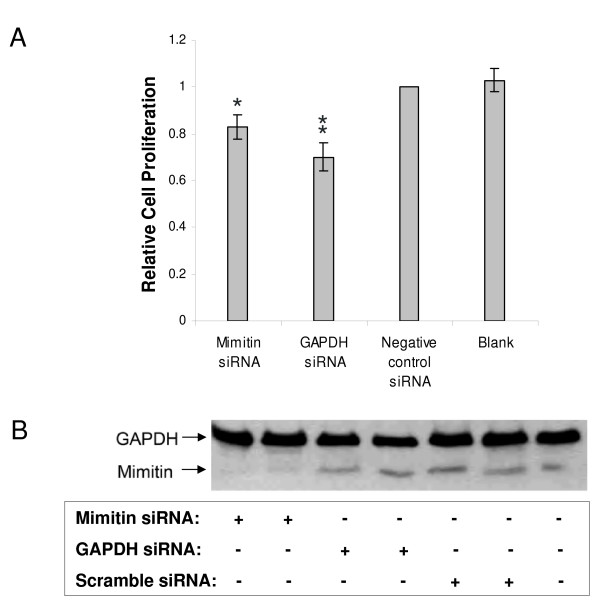
**Effect of mimitin gene silencing on proliferation of HepG2 cells**. Cells were transfected with silencing RNA specific for mimitin (50 nM), or for GAPDH (100 nM, positive control), or unspecific siRNA (negative control, 50 nM) or were treated with transfection reagent siPORT NeoFX alone (blank). At 72 h post transfection cells were incubated with BrdU (5 μM) for 3 h and incorporation was determined with Cell Proliferation ELISA. A. Cell proliferation is shown relative to the non-sepcific siRNA (control). Values were calculated from absorbance readings at 450 nm and represent the mean of three independent experiments with standard deviation (*p < 0.05, **p < 0.02 compared to control). B. Representative Western blot analysis with 20 μg protein from simultaneously seeded 12-well plates using anti-GAPDH or anti-mimitin antibodies.

To exclude the possibility that the observed changes in cell viability are not caused by cytotoxic effects of TNF and CHX, the release of LDH from the cells was measured. It was found that neither transfection of cells with different siRNAs, nor TNF/CHX treatment, lead to increased LDH release which is regarded as an indicator of cell death via necrosis (data not shown). Then the effect of the mimitin gene silencing on cell proliferation was determined by BrdU-incorporation in HepG2 cells transfected with siRNA targeting mimitin. As shown in Fig. 8, silencing with mimitin-A siRNA resulted in a small but significant (*p *< 0.05) decrease of BrdU-incorporation compared to control cells. Thus, we conclude that a reduced cellular level of mimitin slightly decreases the rate of DNA replication and cell proliferation. On the other hand, overexpression of mimitin seemed to have no effect on HepG2 proliferation (data not shown).

## Discussion

Proinflammatory cytokines modulate expression of several mitochondrial proteins participating in ATP generation, as it was shown in our previous studies [3,15]. Cytokines such as IL-1 and IL-6 are known to influence energy metabolism and mitochondrial function [16], significantly inhibiting ATP production and utilization in a time- and dose-dependent manner, as it was shown in primary cultures of rat hepatocytes [17,18]. In our experiments we observed an increase in the levels of mimitin transcript and mimitin protein after 12 and 18 h of HepG2 cells exposure to IL-1 and IL-6. These cytokines also stimulated expression of the luciferase reporter gene under control of the mimitin gene promoter (data not shown). Therefore, it can be concluded that both cytokines regulate mimitin gene expression at the transcriptional level.

It is puzzling why the proinflammatory cytokines activate expression of a gene coding for a mitochondrial protein. In an attempt of answering this question we looked for proteins interacting with mimitin. Using yeast two-hybrid system and co-immunoprecipitation we found that one of the proteins interacting with mimitin is MAP1S. This is a recently described member of the microtubule-associated protein 1 family, expressed in a wide range of tissues and synthesized as a precursor protein that is cleaved into heavy and light chains in a tissue-specific manner [19]. Liu and co-workers [9] showed that MAP1S is complexed with mitochondrial NADH dehydrogenase I and cytochrome c oxidase I resulting in "mitochondrial aggregation and genome destruction" [8]. The results of Liu and co-workers suggest that MAP1S mediates communication between the microtubular cytoskeleton and mitochondria, especially in control of cell death [8].

Taking into consideration that mimitin is a mitochondrial protein and that it probably functions as a molecular chaperone essential for the efficient assembly of complex I [5], we expected that this protein could be important for cell viability. To investigate this hypothesis we studied the effect of either inhibition or overexpression of mimitin on cell proliferation, and cell survival. Blocking the expression of mimitin in HepG2 cells by siRNA demonstrated that mimitin itself has no direct influence on caspase 3/7 activities. However, when apoptosis was induced with TNF and CHX the activity of caspases was significantly increased by silencing mimitin expression. Conversely, overexpression of mimitin in cells induced to undergo apoptosis reduced the activity of these caspases. Moreover, inhibition of mimitin gene slightly decreased the proliferation rate of HepG2 cells, while mimitin overexpression apparently had no effect.

Treatment of HepG2 cells with TNF/CHX initiates the extrinsic apoptotic pathway and strongly stimulates effector caspases [20,21]. Since overexpression of mimitin leads to a significant decrease in the activites of caspases 3/7 it is possible that potential antiapoptotic properties of mimitin become apparent after interaction with some pro-apoptotic proteins, such as the microtubular protein – MAP1S. Further studies are required to elucidate the significance of these interactions and to establish the exact position of mimitin in cell death-related processes.

## Conclusion

We confirmed that mimitin is a mitochondrial protein and showed that it is regulated by proinflammatory cytokines with a highest basal expression in the heart. MAPK kinases, but not the NFκB-related pathway, were shown to play a pivotal role in IL-1- dependent regulation of mimitin gene. Mimitin was found to interact with the microtubular protein MAP1S and modulation of mimitin gene expression affected the activities of caspases 3 and 7 but only in the cells stimulated to develop apoptosis.

## Methods

### Cell culture and stimulation with IL-1 and IL-6

HepG2 cells (ATCC) were cultured at 37°C and 5% CO_2 _in Dulbecco Modified Eagle Medium (DMEM) with 1000 mg/L D-glucose (Sigma-Aldrich), supplemented with 10% foetal bovine serum (Gibco/BRL). Cells were grown to approximately 80% confluence. Fifteen hours before cytokine treatment they were washed twice in PBS and placed in DMEM containing 0.5% FBS. Then the cells were stimulated with human recombinant IL-1 (BioMol) at the final concentration of 15 ng/ml, or with human recombinant IL-6 (MP) at the final concentration of 25 ng/ml, or with a mixture of both. In the case of experiment with IL-1 or IL-6 applied separately, cells were stimulated for 4, 12, and 24 h. When both cytokines were used, cells were prestimulated for 3 h with IL-1 and then stimulated with IL-6 for the time indicated. The concentration of both cytokines was optimized experimentally. The responsiveness of HepG2 cells to these cytokines was evaluated in each series of experiments by comparing changes in the levels of mRNA coding for known acute phase proteins [3]. Non-stimulated cells harvested after 4, 12, and 24 h of the experiment served as controls.

### Generation of genetic constructs and transfection procedure

The insert for pcDNA3mim and pQE31mim vector, containing complete coding region of mimitin gene, was generated by PCR in two steps. The first round of PCR was carried out with forward primer: tgctggcagcgctggaaac and reverse: agtcacatccatatacatgaaaag. The following nested primers: forward: cggcatgggttggtctc with restriction site for KpnI and reverse: atgcattcattgattgtcgc with restriction site for SmaI/XmaI were used to obtain a region of 520 nucleotides, corresponding to positions -4 to 516 relative to the mimitin ATG start codon. After digestion of the PCR product with KpnI/SmaI and of the pcDNA3 vector with KpnI/EcoRV ligation was carried out. For the recombinant protein purification the same procedure was used but the forward primer had SacI restriction site. Both constructs were sequenced before the transfection/transformation experiment. Plasmids overerexpressing MAP1S were kindly provided by Prof. Friedrich Propst [19]. Expression of MAP1S was under the control of tet-responsive promoter. For overexpression of MAP1S in HepG2 cells co-tranfection of UHD15-1 (containing tTA transactivator) and pUHD10-3 (containing full length MAP1S) was carried out. To overexpress mimitin or MAP1S, HepG2 cells were transfected with the following genetic constructs: pcDNA3 empty vector (control), pcDNA3mim, pUHD15-1 or pUHD10-3 using Lipofectamine 2000 (Invitrogen) according to the manufacturer's procedure.

To estimate the efficiency of transfection in different samples we performed RT-PCR using primers specific for a transcript coding for aminoglycoside phosphotransferase (neomycin resistance gene; coded by pcDNA3) and EF2 as a reference gene (NeoF: tgctcctgccgagaaagtat and NeoR: aatatcacgggtagccaacg; EF2 11: gacatcaccaagggtgtgtgcag and EF2 22: gcggtcagcacaatggaata).

### Northern blot analysis

In order to analyse tissue distribution of the mimitin transcript a commercial blot filter was used (Clontech). The membrane had been loaded with 2 μg of mRNA isolated from the following human tissues: brain, heart, skeletal muscles, colon, thymus, spleen, kidney, liver, small intestine, placenta, lung and peripheral blood leukocytes. Prehybridization and hybridization were carried out at 65°C in 1% SDS, 1 M NaCl, 10% dextran sulfate solution. Thirty nanograms of the probe was labelled with [α-^32^P]dCTP using a random primer labelling kit (Promega). After washing (20 min at room temperature in 2 × SSC, 20 min at 65°C in 2 × SSC and then twice for 20 min. at 65°C in 1 × SSC/1% SDS) the RNA blot was read using a Molecular Imager FX and analysed with Quantity One (Biorad). The blot was later stripped and reprobed with a β-actin probe provided with the kit to check for equal loading.

In the experiment where the changes in mimitin transcript level were studied total RNA was isolated from control (unstimulated) cells and from cells after IL-1 and IL-6 treatment. The guanidinum/phenol extraction procedure was used. Ten micrograms of total RNA was separated in 1% agarose-formaldehyde gel and blotted onto a nitrocellulose membrane. Prehybridization and hybridization were carried out as above. Quantitative analysis of the transcript was based on densitometric readings from Quantity One software. The intensity of each analysed band was divided by the intensity of the 28S rRNA band and the final results were calculated as the multiple of suitable control. The reported values represent the mean of three independent experiments.

### Generation of mimitin-specific monoclonal and polyclonal antibodies

*E. coli *BL21 cells were transformed with pQE-31 vector containing the entire coding sequence of mimitin. Recombinant protein was purified from bacterial culture using BD Talon Metal Affinity Resins with cobalt ions (BD Biosciences) and used in immunization of New Zealand White rabbit (3 times 300 μg of antigen). Antibodies were purified from rabbit sera by protein A-Sepharose chromatography and then checked for specificity. The antibodies were analyzed with protein extracts from HepG2 cells and with recombinant mimitin by Western blotting. There was no cross-reactivity and only specific product of the expected size was observed on the membrane. An additional proof of the quality and specificity of the obtained antibodies is the observed reduction of the signal in the Western blot after the cells were transfected with siRNA specific for mimitin.

For generation of a monoclonal antibody Balb/c mice were immunized several times with recombinant mimitin according to a standard protocol [22]. The specific IgG titre exceeded 1:10^5^. Splenocytes were fused with Sp2/0 myeloma cells (HPRT^-/-^) using polyethylene glycol (Sigma-Aldrich) and the mixture of cells was cultured for two weeks in HAT selection medium enriched with 10% FCS and hybridoma growth supplement (Sigma-Aldrich). The clones were analyzed for the synthesis of anti-mimitin Ab in direct ELISA on microplates covered with recombinant mimitin (2 μg/ml). The clone 1F5D8 obtained after a two-step subcloning procedure performed by serial dilution was chosen for further work. Undiluted culture medium of this clone (DMEM + 10%FCS) was used as a source of the detecting antibody in indirect ELISA.

### Cell lysate preparation and Western blot analysis

Whole cell protein extracts were obtained by washing cells twice with ice-cold PBS and harvested in 1 ml of PBS containing 10% (v/v) glycerol and EDTA (5 mM). After centrifugation (1000 g, 10 min, 4°C), cell pellets were suspended in 50 μl of lysis buffer (100 mM Tris-HCl pH 7.5; 1% Triton-X100 and protease inhibitor cocktail (Roche)). Protein concentration of each sample was determined using the bicinchoninic acid assay (BCA, Sigma-Aldrich). Ten micrograms of protein extract was loaded onto 10% polyacrylamide gel and separated by SDS/PAGE in Tris-tricine buffer [23]. After transfer to nitrocellulose membrane blots were blocked with 2% BSA in TBS-N buffer (20 mM Tris pH 8.0, 150 mM NaCl, 0.05% Nonidet P-40) overnight at 4°C and then incubated with primary antibodies for 2 h at room temperature. Polyclonal antibodies against mimitin, generated as described above, were used at 3 μg/ml. Secondary HRP-conjugated anti-rabbit IgG was used at 1:80 000 dilution (Sigma-Aldrich). Both primary and secondary antibodies were diluted with TBS-N containing 2% BSA. TBS-N was used for washing the membranes before, between and after incubation with antibodies. Immunoreactive bands were detected by an enhanced chemiluminescence system (SuperSignal West Pico Chemiluminescent Substrate, Pierce) and visualized on medical x-ray film.

### Identification of signalling pathway

HepG2 cells stably transfected with retroviral vector pCFG5-IEG2 encoding a non-degradable mutant form of IκBα, and cells with an empty vector (control) were used to determine of the NFκB signalling pathway in IL-1-dependent activation of mimitin gene. These cells were kindly provided by Professor Stephan Ludwig (Heinrich-Heine University, Duesseldorf, Germany; [24]).

For determination of MAPK signalling pathway involvement in mimitin gene activation HepG2 cells were serum-starved overnight in 0.5% FBS in DMEM. Then the cells were pre-treated with MAPK inhibitors: 10 μM U0126 (Calbiochem), 10 μM SP600125 (Calbiochem), or 10 μM ZM336372 (Calbiochem) for 30 min and stimulated with15 ng/ml IL-1 for 12 h. Mimitin transcript level was measured by real time PCR. The inhibitors' activity was controlled by analysis of phosphorylation of MAP kinases (Western blot). For control experiment cells were also pre-treated with the inhibitors and stimulated with 15 ng/ml IL-1 for 10 min. Cell lysates were analyzed by Western blotting with anti-P-Erk and anti-P-JNK (Cell Signaling Technology) and anti-P-p38 antibodies (Abcam).

### Real Time PCR (Q-RT-PCR)

cDNA was synthesized from 1 μg of total RNA in 20 μl using SuperScript RNaseH^- ^reverse transcriptase (Promega) and oligo (dT) primer (Promega). Real time PCR was carried out using the SYBR Green PCR Master Mix (DyNAmo™ HS SYBR Green qPCR (Finnzyme), 1 μl of 5× diluted cDNA, and 20 ng of each primer. For mimitin transcript the forward primer was 5'-ctgcctccaccagttcaaactc-3' and the reverse primer was 5'-gtcgcatccatatacgtgaaaag-3'. The amount of each transcript, expressed as fold-variation over control (untreated cells), was calculated after determination of the difference between the C_T _of the transcript studied and the calibrator transcript for elongation factor 2 (EF2). The C_T _values used were means of triplicate measurements.

### Subcellular localization of mimitin

For subcellular fractionation of HepG2 cells the Qproteome Kit (Qiagen) was used according to manufacturer's procedure. Consecutive centrifugation and extraction with suitable buffers allowed the isolation of cytosolic, membrane, nuclear and cytoskeletal proteins. Protein concentration was determined with BCA protein assay. Additionally, the mitochondrial fraction was isolated as described by Ndubuisi and co-workers [7]. Ten micrograms of protein extracts from each fraction was used in Western blot analysis. For detection of individual proteins different polyclonal antibodies were used (Abcam): for the nuclear fraction – against laminin, for the cytosolic fraction – against GAPDH, for the membrane fraction – those against prohibitin.

### ELISA

Mimitin levels in control and cytokine-stimulated HepG2 cells were measured using sandwich ELISA. A 96-well microtiter plate was coated with rabbit polyclonal antibodies against mimitin. To each well 100 μl of antibodies (6 μg/ml) diluted in 50 mM borate buffer (pH 8.35) was added. Plates were incubated overnight at 4°C. After washing five times with PBS a protein standard (6, 12, 25, 50, 100, 200, 400, 800 pg/ml in 0.5% BSA in PBS) and cell lysates (2.5 μg) were added in duplicates. Incubation for 1 h at 37°C was followed by washing with PBS to remove unbound proteins. Subsequently, plates were incubated with biotin-labelled anti-mimitin antibodies (1 μg/ml in 0.5% BSA in PBS) for 1 h at 37°C. Then, to each well 50 μl of avidin-HRP (Sigma) (1:25000 in 0.5% BSA in PBS) was added and incubation was carried out for 30 min at 37°C. Each incubation step was followed by extensive washing with PBS. The enzymatic reaction was performed using the TMB Substrate Reagent Set (BD Biosciences), terminated by adding 50 μl 1 M HCl and the absorbance was measured at 450 nm. The values represent the mean ± SD of three independent experiments performed in duplicates.

### siRNA transfection

Pre-designed siRNA obtained from Ambion included GAPDH siRNA as a positive control, and siRNA with scrambled sequence as a negative control. The two different siRNA sequences specific for mimitin were as follows: (A) gaaaucaaaauaaaaagccuu and (B) gccacaaucaaugaaugcatt.

HepG2 cells were cultured in 12-well plates as described above. The siRNA concentration and the amount of transfection reagent were optimized experimentally. Before transfection, cells were trypsinized, centrifuged at 1000 rpm at 4°C for 5 min and resuspended in fresh medium. Lipid-based transfection reagent siPORT NeoFX (4 μl/well) from Ambion and siRNAs (50 nM final concentration) were separately diluted in 50 μl OPTIMEM and then mixed together. Transfection complexes were allowed to form for 10 min and overlayed with 9 × 10^4 ^cells per well. As an additional control, some wells were treated with transfection reagent only. The silencing of mimitin was confirmed by Western blotting in three independent experiments.

### Caspase activity assay

To analyse caspase 3/7 activity, HepG2 cells were transfected with mimitin-specific siRNA, GAPDH-specific siRNA or negative control siRNA as described before. After 72 h incubation, apoptosis was induced in some of the wells with 15 ng/ml TNFα (PromoCell) and cycloheximide at the final concentration of 40 μM per well. Cells were scraped in RIPA buffer (25 mM Tris HCl pH7.6, 150 mM NaCl, 1% NP-40, 1% sodium deoxycholate, 0.1% SDS) when most of the stimulated cells in a well showed signs of apoptosis before complete detachment (after 5 h of treatment). Apoptosis was assessed by analysis of activation of caspase-3 and -7 using the DVD-aminoluciferin substrate from the Caspase-GloTM3/7 assay kit (Promega). After protein isolation, 5 μg of protein from each well was diluted in RIPA buffer to a final volume of 50 μl and added to an equal amount of Glo-reagent. Reaction mixture was incubated for 1 h at room temperature in the dark. Caspase 3/7 activity was measured using luminometer (MicroLumat LB96P; EG&G Berthold).

### BrdU-Incorporation

HepG2 cells were transfected with siRNA specific for mimitin, GAPDH or negative control siRNA in a 96-well plate. In each well 800 cells were seeded and the ratio of transfection mixture to cell density was the same as in previous experiments. Additionally, cells were transfected in a 12-well plate for Western blot analysis. BrdU-labelling solution from the Cell Proliferation ELISA kit (Roche Applied Sciences) was diluted 1:2000 and added to the 96-well plate 54 h post transfection. After 24 h of labelling, protein fraction was isolated from 12-well plates and medium was removed from 96-well plates. Incorporated BrdU was detected via ELISA with anti-BrdU-POD antibody diluted 1:200 according to the manufacturer's protocol.

### Yeast two-hybrid analysis

For yeast two-hybrid screening the Matchmaker human foetal brain cDNA library was used according to manufacturer's procedure (Clontech). The 510-bp fragment encoding full length cDNA of mimitin was generated by RT-PCR using primers:

F – atgggttggtctcagga with restriction site for NdeI, and R-atgcattcattgattgtggct with restriction site for BamHI. After digestion with NdeI/BamHI the PCR fragment was cloned into pGBKT7 vector of the same restriction sites. The correctness of the cDNA sequence was confirmed by sequencing. Before the cDNA library screening, two control experiments were carried out: to verify whether the pGBKT7-mimitin vector expresses the protein in the AH109 strain of *Saccharomyces cerevisiae*, and to find out whether the "bait" (mimitin) does not autonomously activate the reporter genes. Expression of the fusion protein Gal4-mimitin was verified by Western blotting with monoclonal antibodies specific for c-Myc. Two-hybrid screening was carried out by mating the AH109 yeast transformed with pGBKT7-mimitin (bait) and Y187 yeast transfected with human foetal brain cDNA library in pACT2 vector. Positive and negative controls for the experiment were carried out in accordance with the protocol. A portion of the mating culture in the appropriate dilution was plated on SD/-trp, SD/-leu and SD/-trp,-leu selection medium to calculate the number of screened cells and determine the mating efficiency. Altogether, we screened 6 × 10^5 ^clones and achieved 8% mating efficiency. The rest of the mating culture was spread on a plate with SD/-trp,-leu,-his,-ade medium to select colonies containing both bait and prey. Plasmids isolated from positive clones were purified from yeast and those containing "prey" were amplified in *E. coli *and co-transformed to yeast with the original bait (pGBKT7-mimitin) to verify specific activation of the reporter genes (HIS3 and lacZ) and the sequences compared with NCBI data.

### Coimmunoprecipitation

HepG2 cells were transfected with the pcDNA3mim plasmid encoding mimitin or with pUHD10-3 encoding Myc-tagged MAP1S and its transactivator pUHD15-1, or co-transfected with all these vectors using Lipofectamine 2000 (Invitrogen). The coimmunoprecipitation analysis was performed 24 h after transfection with the ProFoundTM c-Myc Tag IP/Co-IP Kit (Pierce) following the manufacturer's protocol. Cell lysates and obtained eluates were loaded on 10% SDS-PAGE for Western blotting. Proteins were detected with antibodies specific for mimitin and for the Myc tag.

### Statistical analysis

The data from real-time PCR, Northern blotting, measurements of caspase activity and of proliferation rate were examined for statistical significance using Student's *t *test. A *p *value less than 0.05 was taken as statistically significant.

## Authors' contributions

PW carried out experiments on cytokine-dependent regulation of mimitin gene expression, coimmunoprecipitation, analysis of signalling pathways, participated in design of experiments and performed statistical analysis. SJY coordinated yeast two-hybrid analysis, contributed to design of the work and critical revision of manuscript. NF carried out experiments with siRNA. AKO substantially contributed to interpretation of data and participated in writing of the manuscript. MB contributed to preliminary studies with siRNA and generated monoclonal antibody. DM carried out Northern blotting and statistical analysis. SM carried out Western blotting. MP carried out Western blotting and ELISA test. AKA performed promoter activation studies. NKT analysed signalling pathways and participated in manuscript revision. JB participated in monoclonal antibody generation and revised the manuscript. JJ generated polyclonal antibody. JOJ designed the experiments and co-ordinated the project, provided interpretation of data, prepared and revised the manuscript. All authors read and approved the final manuscript.

## References

[B1] Baumann H, Gauldie J (1994). The acute phase response. Immunol Today.

[B2] Koj A, Jura J (2003). Complex analysis of genes involved in the inflammatory response: interleukin-1-induced differential transcriptome of cultured human hepatoma HepG2 cells. Acta Biochim Pol.

[B3] Wegrzyn P, Jura J, Kupiec T, Piekoszewski W, Władyka B, Zarebski A, Koj A (2006). A search for genes modulated by interleukin-6 alone or with interleukin-1beta in HepG2 cells using differential display analysis. Biochim Biophys Acta.

[B4] Tsuneoka M, Teye K, Arima N, Soejima M, Otera H, Ohashi K, Koga Y, Fujita H, Shirouzu K, Kimura H, Koda Y (2005). A novel Myc-target gene, mimitin, that is involved in cell proliferation of esophageal squamous cell carcinoma. J Biol Chem.

[B5] Ogilvie I, Kennaway NG, Shoubridge EA (2005). A molecular chaperone for mitochondrial complex I assembly is mutated in a progressive encephalopathy. J Clin Invest.

[B6] Lazarou M, McKenzie M, Ohtake A, Thorburn DR, Ryan MT (2007). Analysis of the assembly profiles for mitochondrial- and nuclear-DNA-encoded subunits into complex I. Mol Cell Biol.

[B7] Ndubuisi MI, Guo GG, Fried VA, Etlinger JD, Sehgal PB (1999). Cellular physiology of STAT3: Where's the cytoplasmic monomer?. J Biol Chem.

[B8] Liu L, Vo A, Liu G, McKeehan WL (2005). Distinct structural domains within C19ORF5 support association with stabilized microtubules and mitochondrial aggregation and genome destruction. Cancer Res.

[B9] Liu L, Amy V, Liu G, McKeehan WL (2002). Novel complex integrating mitochondria and the microtubular cytoskeleton with chromosome remodeling and tumor suppressor RASSF1 deduced by in silico homology analysis, interaction cloning in yeast, and colocalization in cultured cells. In Vitro Cell Dev Biol Anim.

[B10] Woo M, Hakem R, Soengas MS, Duncan GS, Shahinian A, Kagi D, Hakem A, McCurrach M, Khoo W, Kaufman SA, Senaldi G, Howard T, Lowe SW, Mak TW (1998). Essential contribution of caspase 3/CPP32 to apoptosis and its associated nuclear changes. Genes Dev.

[B11] Zheng TS, Schlosser SF, Dao T, Hingorani R, Crispe IN, Boyer JL, Flavell RA (1998). Caspase-3 controls both cytoplasmic and nuclear events associated with Fas-mediated apoptosis in vivo. Proc Natl Acad Sci USA.

[B12] Degterev A, Boyce M, Yuan J (2003). A decade of caspases. Oncogene.

[B13] Kim JW, Kim TE, Kim YK, Kim YW, Kim SJ, Lee JM, Kim IK, Namkoong SE (1999). Antisense oligodeoxynucleotide of glyceraldehyde-3-phosphate dehydrogenase gene inhibits cell proliferation and induces apoptosis in human cervical carcinoma cell lines. Antisense Nucleic Acid Drug Dev.

[B14] Norris AJ, Sartippour MR, Lu M, Park T, Rao JY, Jackson MI, Fukuto JM, Brooks MN (2008). Nitroxyl inhibits breast tumor growth and angiogenesis. Int J Cancer.

[B15] Jura J, Wegrzyn P, Zarebski A, Władyka B, Koj A (2004). Identification of changes in the transcriptome profile of human hepatoma HepG2 cells stimulated with interleukin-1 beta. Biochim Biophys Acta.

[B16] Zell R, Geck P, Werdan K, Boekstegers P (1997). TNF-alpha and IL-1 alpha inhibit both pyruvate dehydrogenase activity and mitochondrial function in cardiomyocytes: evidence for primary impairment of mitochondrial function. Mol Cell Biochem.

[B17] Kitade H, Kanemaki T, Sakitani K, Inoue K, Matsui Y, Kamiya T, Nakagawa M, Hiramatsu Y, Kamiyama Y, Ito S, Okumura T (1996). Regulation of energy metabolism by interleukin-1beta, but not by interleukin-6, is mediated by nitric oxide in primary cultured rat hepatocytes. Biochim Biophys Acta.

[B18] Berthiaume F, MacDonald AD, Kang YH, Yarmush ML (2003). Control analysis of mitochondrial metabolism in intact hepatocytes: effect of interleukin-1beta and interleukin-6. Metab Eng.

[B19] Orbán-Németh Z, Simader H, Badurek S, Tranciková A, Propst F (2005). Microtubule-associated protein 1S, a short and ubiquitously expressed member of the microtubule-associated protein 1 family. J Biol Chem.

[B20] Boatright KM, Salvesen GS (2003). Mechanisms of caspase activation. Curr Opin Cell Biol.

[B21] McDonnell MA, Wang D, Khan SM, Heiden MG Vander, Kelekar A (2003). Caspase-9 is activated in a cytochrome c-independent manner early during TNFalpha-induced apoptosis in murine cells. Cell Death Differ.

[B22] Peters J, Baumgarten H (1992). Monoclonal Antibodies: A Practical Guide.

[B23] Schagger H, von Jagow G (1987). Tricine-sodium dodecyl sulfate-polyacrylamide gel electrophoresis for the separation of proteins in the range from 1 to 100 kDa. Anal Biochem.

[B24] Wurzer WJ, Ehrhardt C, Pleschka S, Berberich-Siebelt F, Wolff T, Walczak H, Planz O, Ludwig S (2004). NF-kappaB-dependent induction of tumor necrosis factor-related apoptosis-inducing ligand (TRAIL) and Fas/FasL is crucial for efficient influenza virus propagation. J Biol Chem.

